# Mpox in East Africa: Confronting a public health emergency

**DOI:** 10.1016/j.jtumed.2024.09.010

**Published:** 2024-10-04

**Authors:** Mohamed Mustaf Ahmed, Najib Isse Dirie, Olalekan John Okesanya, Don Eliseo Lucero-Prisno

**Affiliations:** a Department of Medicine and Surgery, Faculty of Medicine and Health Sciences, SIMAD Univeristy, Mogadishu, Somalia; b Department of Urology, Dr. Sumait Hospital, Faculty of Medicine and Health Sciences, SIMAD University, Mogadishu, Somalia; c Department of Public Health and Maritime Transport, University of Thessaly, Volos, Greece; d Department of Global Health and Development, London School of Hygiene and Tropical Medicine, London, United Kingdom; e Department of Research and Development, Biliran Province State University, Naval, Philippines; f Department of Research and Innovation, Southern Leyte State University, Sogod, Philippines

Dear Editor,

The recent resurgence of Mpox, particularly its impact on Africa, highlights existing health disparities and regional vulnerabilities. While the World Health Organization (WHO) declared Mpox a Public Health Emergency of International Concern (PHEIC) in July 2022, the escalating situation in Africa, especially East Africa, underscores the troubling trend of rising cases amidst constrained resources.^1^ In response to a new outbreak in August 2024, WHO issued another PHEIC, which was triggered by a different clade of the virus with a higher mortality rate, particularly affecting East Africa.^2^ Although Mpox was initially concentrated in Central and West Africa, it has shown the ability to evolve rapidly and extend its geographical reach. This is especially troubling in East Africa, where healthcare infrastructure and disease surveillance are notably limited.^3^ Historically, East Africa has reported lower incidence rates than other parts of the continent; however, the current emergence of Mpox in this region now represents a critical public health emergency. The surge in cases can be partly attributed to the expanding outbreak and enhanced national responses, which have led to increased testing and confirmation of cases, particularly in the Democratic Republic of the Congo (DRC). In July and August 2024, four East African countries—Burundi, Kenya, Rwanda, and Uganda—reported their first-ever Mpox cases.^3^ Genomic sequencing confirmed that these cases were predominantly linked to clade I of the Mpox virus, tracing the origins of the outbreak back to the expanding crisis in East and Central Africa.^3^ The severity of this situation led the Africa Centers for Disease Control and Prevention (Africa CDC) to declare the Mpox outbreak a Public Health Emergency of Continental Security on August 13, 2024.^4^ This declaration underscored the urgent need to mobilize resources and strengthen collaborative efforts to control the spread of the virus.

Data from WHO have revealed a pattern of rising infections. From January 1, 2022, to June 30, 2024, a total of 99,176 laboratory-confirmed Mpox cases were reported across 116 countries, resulting in 208 deaths.^3^ In June 2024, WHO reported 934 new Mpox cases globally, with the African region accounting for 61 % of these cases. This marked the second consecutive month in which Africa confirmed more cases than any other region.^3^ While this is partially due to improved national responses, including expanded testing in the DRC, the surge also signifies an expansion of the outbreak within the continent. These trends directly impact East Africa, as evidenced by the emergence of Mpox in four East African countries.^5^ The cases in Burundi, Kenya, Rwanda, and Uganda, confirmed through genomic sequencing to be predominantly clade I of the Mpox virus, highlight the interconnectedness of this public health crisis in Africa and the particular vulnerability of East Africa within this evolving epidemiological landscape.^5^

East African nations face significant challenges in combating this public health crisis effectively. The region struggles with limited access to diagnostics, hindering early detection and timely intervention.^6^ Healthcare infrastructure remains fragile, particularly in rural areas where the capacity to manage surges in Mpox cases is severely limited. Compounding these challenges is the scarcity of vaccines and therapeutics.^7^ During the 2022 global outbreak, most of these vital resources were directed toward high-income nations, leaving African countries with limited access. This inequitable distribution of resources exacerbates existing vulnerabilities and hinders the ability to mount an effective response.^8^ Additionally, concurrent public health threats, such as HIV, further complicate this situation. Addressing these challenges requires a multipronged approach that addresses both the immediate needs of the outbreak response and the underlying systemic weaknesses that exacerbate its impact.

Global and continental organizations have mobilized resources in response to the escalating Mpox crisis in East Africa. The WHO elevated the global Mpox event to an acute grade 3 emergency, triggering the release of funds from the Contingency Fund for Emergencies (CFE) to strengthen response efforts in Africa.^3^ The organization is collaborating with national and local health authorities, health workers, civil society, and partners to develop strategies to increase access to diagnostics, enhance clinical care and access to vaccines, ensure stigma-free risk communication and community engagement, and reinforce global preparedness and response efforts to contain Mpox effectively both locally and globally.^3^ The organization is also working toward securing emergency use listings for Mpox vaccines, aiming to expedite their availability in affected regions, including supporting outbreak response in East African countries.^9^ The Africa CDC is also working to secure 200,000 doses of the Mpox vaccine from Bavarian Nordic, a biotechnology firm in Hellerup, Denmark, to help stop the outbreak. The Africa CDC estimates that ten million doses are required to prevent a current outbreak.^8^ A key aspect of the response involves reinforcing laboratory testing to comprehend the true scale of the outbreak and guide effective interventions.^4^

In conclusion, the emergence of Mpox in East Africa poses a significant threat to the fragile healthcare systems of the region and underscores the need for swift and decisive action. This situation necessitates an immediate and coordinated response from regional and global health authorities. The emergence of Mpox in countries such as Burundi, Kenya, Rwanda, and Uganda, which were previously unaffected by the virus, highlights the urgent need for increased vigilance and proactive measures to prevent further spread within the African continent. These sources emphasize that the reported case counts likely represent a significant underestimation due to limitations in diagnostic capacity and surveillance systems. This underscores the need for the creation of Africa's *New Public Health Order*, comprising five strategic pillars—workforce development, enhanced vaccine production, diagnostics and therapeutics, domestic health security resources, and action-oriented partnerships—to be invested in the continent. This will help Africa move away from a global health model that is centered on charity and aid and toward one that is based on independence and self-sufficiency. The continent needs to produce diagnostics, medications, and vaccinations in addition to having access to cutting-edge technology like the mRNA platform. A data-driven approach is vital for informing targeted public health interventions and resource allocation strategies tailored to the specific challenges faced in East Africa.

## Source of funding

The authors have not received any funding for this study.

## Ethical approval

Not applicable.

## Author contributions

MMA and NID conceptualized and designed the study. OJO conducted literature review and data collection. MMA wrote the first draft of the manuscript. All the authors critically revised the manuscript for important intellectual content. DELP III supervised the study. All authors have read and approved the final manuscript.

## Conflict of interest

The authors have no conflict of interest to declare.
